# Development and Design of a Pediatric Case-Based Virtual Escape Room on Organophosphate Toxicity

**DOI:** 10.21980/J8DH1V

**Published:** 2024-07-31

**Authors:** Kaitlyn Boggs, Manu Madhok, Tania Ahluwalia

**Affiliations:** * Medical University of South Carolina, Department of Emergency Medicine, Charleston, SC; ^ Children’s Minnesota, Department of Emergency Medicine, Minneapolis, MN; † Children’s National Hospital, Department of Emergency Medicine, Washington, DC; ** George Washington University, Washington, DC

## Abstract

**Audience:**

This case-based virtual escape room (VER) serves as a didactic activity suitable for learners who require an understanding of organophosphate toxicity. Educators may use this VER for distance-based learning in settings with adequate internet access.

**Introduction:**

India faces a concerning escalation in suicide rates, particularly among teenagers and young adults, often involving intentional pesticide ingestion, notably organophosphates.^1^–^3^ Our project addresses organophosphate ingestion by using a VER, a virtual learning platform adapted from in-person escape rooms to engage participants for educational purposes.^4^,^5^ Demonstrating success in medical, pharmacy, and nursing education, VERs increased satisfaction and competency among healthcare trainees compared to traditional learning platforms while fostering teamwork and communication in a virtual learning environment.^6^,^7^

**Educational Objectives:**

By the end of the activity, learners should be able to: 1) recognize risk factors, symptoms, and presentation for organophosphate poisoning; 2) understand the radiologic and laboratory findings in organophosphate poisoning; 3) distinguish and differentiate electrocardiogram findings in common toxic ingestions; 4) explain the pathophysiology of organophosphate poisoning; 5) understand the importance of decontamination of the patient and personal protective equipment for staff for organophosphate poisoning; 6) describe the airway management of organophosphate poisoning; 7) describe the medical management of organophosphate poisoning, including antidotes and the correct dosing and 8) demonstrate teamwork through communication and collaboration.

**Educational Methods:**

The development process involved a seven-step approach, beginning with topic selection. The process involved creating a scenario, defining learning objectives, and designing an appropriate room. Clues and puzzles were tailored to align with the learning objectives and promote interactivity. The VER was hosted on Google Sites (Google LLC), accompanied by a facilitator guide offering content and technical support.

**Research Methods:**

This VER leverages technology for distance learning, using Zoom (Zoom Video Communications Inc.) for online sessions with EM trainees. Participants were organized into small groups in breakout rooms on Zoom, following a structured format that included a pre-briefing, a timed escape room scenario, debriefing, and evaluation. Afterward, an evaluation in the format of a survey was distributed to participants. This study was Institutional Review Board exempt.

**Results:**

Out of 120 participants in the VER, 50% responded to a survey. The majority found the activity interactive, engaging, and exciting. This feedback indicated a positive reaction to the VER, consistent with the Kirkpatrick model’s first level of assessment.^8^ The VER effectively promoted learning and reinforced clinical knowledge, contributing to the second level of the Kirkpatrick model. In this case, 84.7% of respondents were able to identify knowledge gaps, and 84.2% of respondents found this to be a feasible model to reinforce medical knowledge.

**Discussion:**

This innovative VER addresses the value of distance-based learning in any setting with an internet connection. It has successfully enhanced collaboration and communication among participants in small groups, making it a valuable resource for medical education. This study has several limitations worth noting including a relatively low survey response rate. Baseline data was not collected prior to the VER. Additionally, the VER was not designed to be an open-book assessment; however, the absence of an in-person moderator makes it challenging to ascertain whether participants used external resources. Furthermore, the exclusive focus of this VER on a single topic may diminish its overall use compared to more traditional didactic sessions. This study is also limited by lack of long-term outcome data. Future studies could further assess knowledge improvement and clinical application. The authors plan to develop additional case-based VERs to advance EM trainees’ knowledge, skills, and communication. Overall, the VER offers a promising and free educational tool for distance learning with potential benefits for various settings with internet access.

**Topics:**

Escape room, gamification, global health, organophosphate poisoning, simulation, virtual escape rooms.

## USER GUIDE

[Table t3-9-3-sg36]

**Table t3-9-3-sg36:** 

List of Resources:	
Abstract	36
User Guide	38
Small Groups Learning Materials	42
Appendix A: Facilitator Guide	42
Appendix B: Key Clinical Pearls	57
Appendix C: Formal Presentation	69
Appendix D: Survey	61

**Learner Audience:**

Interns, Junior Residents, Senior Residents, Global Health Educators, Simulation Educators

**Time Required for Implementation:**

We modeled our VER sessions similar to tele-simulations with a pre-brief, followed by a scenario, debrief, education, and evaluations. The initial ten minutes oriented the learners to the platform and organized them into small groups. Next, a 75-minute timer started, and participants were presented with a 13-year-old patient’s chief complaint and initial presentation. The case then progressed through a physical exam, followed by medical and social history. Puzzles provoked thoughts regarding differential diagnoses, and then participants worked through the management of the case. Clues reviewed the pathophysiology, decontamination, and medical management of the suspected toxin. As the VER progressed, the patient decompensated, and a final clue was presented. Participants were required to demonstrate their knowledge about the antidotes to escape the room, and ultimately, save the patient within the 75-minute timeframe. After this time marker, regardless of the participants’ success in escaping the room, a 15-minute debrief was conducted, followed by education.

**Recommended Number of Learners per Instructor**:

The session was led over two separate occasions to accommodate all participants. All trainees were randomized via Zoom’s breakout room function into small teams and reviewed to ensure each group contained learners of all postgraduate years. A breakout room included six to ten participants and one dedicated facilitator for support and technical assistance. Cameras were requested to be on for all participants with a closed-book format teaching session. This VER design allows future participants to engage as an in-person group, via video conferencing platforms or asynchronous learning.

**Topics:**

Escape room, gamification, global health, organophosphate poisoning, simulation, virtual escape rooms.

**Objectives:**

By the end of the activity, learners should be able to:

1. Recognize risk factors, symptoms, and presentation for organophosphate poisoning
2. Understand the radiologic and laboratory findings in organophosphate poisoning
3. Distinguish and differentiate electrocardiogram findings in common toxic ingestions
4. Explain the pathophysiology of organophosphate poisoning
5. Understand the importance of decontamination of the patient and personal protective equipment for staff for organophosphate poisoning
6. Describe the airway management of organophosphate poisoning
7. Describe the medical management of organophosphate poisoning, including antidotes and the correct dosing
8. Demonstrate teamwork through communication and collaboration

### Linked objectives and methods

We established a seven-step process for creating our VER (Table 1), utilizing a facilitator guide to organize each step. First, we identified the high-yield topic of organophosphate toxicity for our learners and designed a case scenario including patient presentation, physical exam, initial workup and evaluation, and acute management. Secondly, distinct learning objectives were defined. Third, we designed a contextually appropriate room based on a real patient room. The VER was formatted on Google Sites, a free platform that allows novice users to customize their websites. We referenced previous work in the education field to structure our VER. Fourth, clues were created to align with learning objectives, including puzzles that added interactive components. Various puzzle choices included word scramble, word search, crossword puzzle, jigsaw puzzle, ciphers, quizzes, and matching games. A key was disguised within each clue to prompt participants to the next clue in the sequence. These clues and keys were generated utilizing Google Forms, Google Slides, and a variety of puzzle-creation websites. Fifth, the Google Site was embedded with these clues for interactivity and simplicity on one webpage. Next, the facilitator guide (Appendix A) was finalized for content and technological support. Finally, a pre-workshop meeting was held with all facilitators to review the content and technical components and was recorded for additional review. Before the activity, the VER underwent trials with faculty members. Pre-brief and debrief techniques were identified, recognizing that VER differs from a traditional simulation.

### Recommended pre-reading for facilitator

Facilitators need sufficient knowledge of organophosphate toxicity. Facilitators were pediatric-emergency medicine trained with interest in simulation. Before the live session, all facilitators attended or reviewed an overview of how the platform worked to understand technological components. They were then encouraged to trial the VER using a link to familiarize themselves with the layout. We recommended reviewing the facilitator guide, covering VER details, clue locations, puzzles, and solutions (Appendix A), along with key clinical pearls and references for further reading (Appendix B). Facilitators were instructed to use a laptop or desktop computer.

### Small group application exercise (sGAE)

Attached as Appendix A

### Results and tips for successful implementation

#### Evaluation

Immediately following the completion of the VER, participants completed an evaluation in the form of a survey including demographics, open-ended feedback, and a 5-point Likert-scale satisfaction rating for medical content and team collaboration. This study included 120 participants, with a 50% response rate (n=60) and equal representation across training years (PGY-1, PGY-2, and PGY-3), as well as equal representation of males and females. Half of the respondents were novices to in-person escape rooms. All groups escaped in the designated time, and the activity was described as “interactive” and “engaging.” Most respondents agreed this was a valuable educational method for medical content (84.2%) and collaborative communication (84.7%). Most perceived the design as easy to follow (81.4%), and participants agreed that the puzzles did not distract from the overall case presentation (81%). This feedback is consistent with the Kirkpatrick model’s positive first-level “Reaction,” which measures whether learners find the training engaging, favorable, and relevant.^8^ Table 2 summarizes participants’ responses, revealing that 86.2% agreed they would partake in future VER experiences.^9^ Many highlighted that they enjoyed the puzzles; one respondent stated it was a “unique approach to broaden their clinical knowledge.” The second level, “Learning,” of the Kirkpatrick Model, which explores new skills, knowledge, and attitudes, was addressed with a debriefing session and evaluation.^8^ The learners’ enthusiastic engagement and willingness to share insights from their clinical practice regarding organophosphate toxicity strengthened the learning objectives and enriched the educational experience. Survey results also provided insights for improvement with recommendations for smaller groups to facilitate agreement on clues, more active participation in the screen-sharing mode, and technological enhancements to reduce internet window pop-ups.^9^

#### Pre-brief

A thorough pre-brief is beneficial to account for variations in participants’ technological understanding and to establish clear logistical rules. As the platform may be novel to users, a comprehensive pre-brief helps minimize technical difficulties during the VER, allowing participants to focus on the educational aspects.

#### Virtual Escape Room

This VER was free to design for distance learning and is accessible in any setting with an internet connection. It is recommended to use a computer, not a tablet or phone, for optimal viewing capability of the VER. Each small group designates a team leader to share their screen and audio. The group members can see the VER via screen-sharing, facilitating communication and collaboration for teammates to solve clues. Alternatively, participants may share one device for in-person collaboration. While a thorough pre-brief is provided, facilitators are essential for addressing potential logistical or technical issues. We recommend facilitators serve as resources if a team encounters technical difficulties; however, we advise them to refrain from providing direct answers, aiming instead to encourage participant’s critical thinking.

#### Debrief

Similar to simulations, prior studies on in-person escape rooms have shown that debriefing enables participants to reflect on teamwork and communication skills while identifying knowledge gaps.^6^,^10^,^11^ We used a standard debriefing format to guide a discussion, prompting participants to reflect, review, and discuss the VER event.^12^,^13^ The Promoting Excellence and Reflective Learning in Simulation **(**PEARLS) debriefing approach combines self-assessment, focused discussion, and teaching to facilitate simulation-based eduation.^13^ We also identified positive team collaborations and discussed areas of improvement. We concluded the debriefing with key take-home points and how this scenario may contribute to their future clinical practice. Following the debriefing, an overview of the learning objectives was provided to ensure all participants understood the topic. This information may be given through the provided handout in addition to a formal presentation (Appendices B and C).

### Associated Content

Appendix A: Facilitator Guide
Appendix B: Key Clinical Pearls
Appendix C: Formal Presentation
Appendix D: Survey

### Pearls

Attached as Appendix B

## Supplementary Information



## Figures and Tables

**Table 1:** How to Design a Virtual Escape Room

Process Steps
1. Identify topic, patient case, and desired audience Begin to format a facilitator guide
2. Define clear learning objectives
3. Select online platform for VER design
4. Create clues, keys, and locks that link to learning objectives
5. Embed clues within the VER online platform
6. Complete facilitator guide to outline patient case, learning objectives, VER design, and progression of clues
7. Define pre-brief and debrief discussion questions

**Table 2:** Participant Evaluation of Case-Based Virtual Escape Room Activity

Question	Agree (%)	Disagree (%)	Neither (%)
The design was easy to follow	81.4	11.8	6.8
*PGY-1*	*28.8*	*1.6*	
*PGY-2*	*30.5*	*5.1*	*6.8*
*PGY-3*	*22.1*	*5.1*	
The addition of puzzles did not take away from the patient case	81	12.1	6.9
*PGY-1*	*31*	*1.7*	*5.2*
*PGY-2*	*31*	*3.4*	*1.7*
*PGY-3*	*19*	*6.9*	
This educational model created a stressful environment	20.7	67.2	12.1
*PGY-1*	*10.3*	*22.4*	*5.2*
*PGY-2*	*5.2*	*24.1*	*6.9*
*PGY-3*	*5.2*	*20.7*	
I would prefer virtual escape room over traditional didactics	73.7	12.3	14
*PGY-1*	*26.3*	*5.3*	*3.5*
*PGY-2*	*26.3*	*3.5*	*7*
*PGY-3*	*21.1*	*3.5*	*3.5*
This was a feasible model to reinforce medical knowledge	84.2	10.5	5.3
*PGY-1*	*29.8*	*3.5*	*3.5*
*PGY-2*	*29.8*	*5.3*	
*PGY-3*	*24.6*	*1.8*	*1.8*
I was able to identify my gaps in knowledge of this case	84.7	8.5	6.8
*PGY-1*	*30.5*	*3.4*	*3.4*
*PGY-2*	*30.5*	*3.4*	*1.7*
*PGY-3*	*23.7*	*1.7*	*1.7*
This utilized critical thinking skills to solve puzzles	80.7	7	12.3
*PGY-1*	*29.8*	*1.8*	*7.1*
*PGY-2*	*28.1*	*3.4*	*3.4*
*PGY-3*	*22.8*	*1.8*	*1.8*
Completing the puzzles facilitated communication between team members	84.7	6.8	8.5
*PGY-1*	*30.5*	*3.4*	*3.4*
*PGY-2*	*23.7*	*1.7*	*3.4*
*PGY-3*	*30.5*	*1.7*	*1.7*

Agree= strongly agree or agree; Disagree= strongly disagree or disagree; Neither= Neither agree or disagree

**Figure f12-9-3-sg36:**
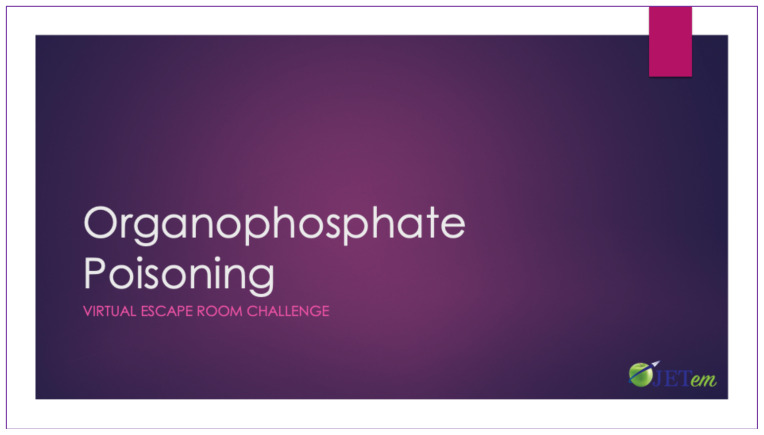
Please see associated PowerPoint file

## SMALL GROUPS LEARNING MATERIALS

### Appendix A Facilitator Guide

#### CASE NARRATIVE

Presentation

A 13-year-old female presents to the emergency department by ambulance with vomiting, diarrhea, confusion, and respiratory distress. Once initial presentation is discussed, the escape room will be opened and the 75-minute timer will start. Participants will learn that the patient lives on a farm with her family and has a history of anxiety, depression and asthma. She is in therapy, followed by a psychiatrist, and on sertraline and amitriptyline daily. Following primary and secondary survey assessment, an ingestion of organophosphates is suspected. The participants will progress through the escape room to evaluate knowledge of the pathophysiology and management of organophosphate poisoning. As management and antidotes are discussed throughout the clues, the patient will have further decompensation, requiring intubation. Once an airway is secured, the final clue will be presented to “escape the room,” unlock the antidote, and ultimately save the patient.

Physical Exam

Vital signs: HR: 52 BP: 79/42 RR: 32 Oxygen: 89%
General: actively vomiting and stooling and ill appearing
HEENT: vision difficulty, miosis, lacrimation, rhinorrhea and drooling
CV: bradycardic, regular rhythm with no murmur or gallop
Resp: In mild respiratory distress with wheezing bilaterally
GI: soft, mild diffuse tender to palpation, hyperactive bowel sounds, no organomegaly
Skin: diaphoretic, lacerations consistent with self-harm on upper extremities
Neuro: confused, complaining of headache

Workup

CXR: Diffuse haziness across both lung fields and left pleural effusion concerning for pulmonary edema.

- WBC: 17 x10^3^/μL
- HGB: 16 g/dL
- Platelets: 235 x10^3^/μL
- Arterial Blood Gas: 7.29/50/53/22/-6
- Na 142 mEq/L
- K 3.8 mEq/L
- Cl 108 mEq/L
- CO2 23 mEq/L
- BUN 11 mg/dL
- Cr 0.8 mg/dL
- Glucose 168 mg/dL
- AST 168 U/L
- ALT 154 U/L
- Amylase 360 U/L

**ESCAPE ROOM:** https://sites.google.com/view/patient-encounter-escape-room/home[Fig f1-9-3-sg36]

**Clue 1 (Patient)**

- Review physical exam by clicking on the patient icon.
  ○ General and neurological exam available on clue 1 webpage
  ○ May assess additional organ symptoms by clicking on face, chest, abdomen respectively
    ▪ Includes audio clips, photographs, and descriptions
- Key to next clue: the patient continues to refer to “her purse” for further history

[Fig f2-9-3-sg36]

**Clue 2 (Purse)**

- Review medical and social history by clicking on the purse icon.
  ○ Past medical history: Depression and anxiety (currently in therapy and followed by psychiatrist), as well as asthma
    ▪ Business card to mental health provider, as well as multiple missed calls from his office
  ○ Current medications: Sertraline, amitriptyline, albuterol
    ▪ Medication bottles noted in her purse
  ○ Social history: Lives on a farm with her parents
    ▪ Photograph of father at their farm, farm background of her phone, text message from family requesting her to pick up fertilizer for the farm today
- Key to next clue: the cipher “xray” highlighted in red letters on the patient’s cellphone[Fig f3-9-3-sg36]

**Clue 3 (X-ray)**

- Word search**:** Participants and facilitator may highlight/annotate words they find via Zoom annotate function.
  ○ Specific and diagnostic for poisoning, but takes time and results should not delay treatment. Should be obtained ideally before antidote is given
    ▪ Answer: RBC cholinesterase
  ○ Rhonchorous breath sounds with imaging findings as seen on chest x-ray
    ▪ Answer: pulmonary edema
  ○ White blood cell count findings possible on CBC
    ▪ Answer: leukocytosis
  ○ Blood gas can show metabolic and/or respiratory****
    ▪ Answer: acidosis
  ○ Can present with pancreatitis-like symptoms
    ▪ Answer: amylase
  ○ Comprehensive metabolic panel can show elevated*****
    ▪ Answer: liver function tests
- Key to next clue: “ekg” highlighted text boxes in yellow after completing the wordsearch[Fig f4-9-3-sg36]

**Clue 4 (EKG)**

- Matching game to compare EKG findings in toxicology.
  ○ Calcium channel blocker: bradycardia + widened QRS[Fig f5-9-3-sg36]
  ○ Antipsychotics (ex: haloperidol) or antidepressants (ex: SSRI) which are potassium channel blockers producing long qt[Fig f6-9-3-sg36]
  ○ TCA overdose (sodium channel blocker): leading to tachycardic + wide QRS + dominant terminal R wave in aVR[Fig f7-9-3-sg36]
  ○ Organophosphate: sinus bradycardia[Fig f8-9-3-sg36]
  ○ Question 5: You suspect your patient has intentionally ingested a toxic substance. After gathering history and assessing the patient, you suspect the patient is presenting with
    ▪ Salicylate toxicity
    ▪ Tricyclic antidepressant toxicity
    ▪ Serotonin syndrome
    ▪ **Organophosphate poisoning**
    ▪ Acetaminophen toxicity
    ▪ Beta blocker toxicity
- Key to next clue: “Look at the monitor”

**Clue 5 (Patient monitor)**

- Drag and drop each presenting symptom to the correct receptor
  ○ Muscarinic receptors at parasympathetic
    ▪ Answer: DUMBELS (Defecation, urination, miosis, bronchorrhea, bronchospasm, bradycardia, emesis, lacrimation, salivation)
  ○ Nicotinic receptors at neuromuscular joint (NMJ)
    ▪ Answer: Fasciculations, muscle weakness, paralysis
  ○ Muscarinic + nicotinic receptors in CNS:
    ▪ Answer: central respiratory depression, lethargy, seizures, coma
- Key to next clue: Once completed, upon clicking “what should you do next?” participants are told to gather their “resuscitation” supplies and photo of code cart.[Fig f9-9-3-sg36]

**Clue 6 (Resuscitation cart)**

- Complete multiple-choice quiz to progress to the next clue.
- In question 4 and 5→the nurse will remind them that she will likely need this dosage later for treatment of the patient. This is because the dosage of atropine is needed to complete the final clue.
- Question 1: Why should pralidoxime (2-PAM) be given as early as possible when organophosphate poisoning is suspected?
  ○ To prevent life threatening bradycardia and cardia arrest
  ○ **To prevent permanent inactivation of the acetylcholinesterase enzyme**
  ○ To prevent the spread of toxicity from the patient to staff
  ○ To prevent respiratory decompensation and intubation
- Question 2: What is true about atropine versus pralidoxime (2-PAM)?
  ○ Pralidoxime is an anti-cholinergic drug that blocks acetylcholine at the muscarinic receptor
  ○ Atropine reversibly binds to the acetylcholinesterase enzyme, competing with the organophosphate binding
  ○ **Atropine will reverse cholinergic symptoms, but will not reverse muscle weakness**
  ○ Pralidoxime is not as effective as atropine at reversing nicotinic effects
- Question 3: Following the initial dose of atropine, how are subsequent doses of atropine titrated?
  ○ The dose is doubled every 3–5 minutes, until resolution of neuromuscular weakness
  ○ The dose is doubled every 3–5 minutes, until resolution of bradycardia
  ○ It is continued as bolus doses until resolution of symptoms and there is no need for maintenance infusion
  ○ The dose is doubled every 3–5 minute**s, until resolution of bronchorrhea and bronchospasm**
- Question 4: What dosage is used of pralidoxime (2-PAM) in children? Remember, the dosage is a range, depending on the severity of the poisoning.
  ○ 100–200 mg/kg via IV
  ○ 0.001–0.003 mg/kg via IV
  ○ 0.02 – 0.05 mg/kg via IV
  ○ **20–50 mg/kg via IV**
- Question 5: What is the initial dosage of atropine in children for organophosphate poisoning? Remember, the dosage is a range, depending on the severity of the poisoning.
  ○ 100–200 mg/kg via IV
  ○ 0.001–0.003 mg/kg via IV
  ○ **0.02 – 0.05 mg/kg via IV**
  ○ 20–50 mg/kg via IV
- Key to the next clue: The nurse points towards the “masks” in the patient’s room.

**Clue 7 (Masks)**

- Word scramble.
  ○ Any staff in contact with the patient should be wearing ____ ____ ____ because the substance is rapidly absorbed through skin, respiratory, and gastrointestinal tract
    ▪ Answer: personal protective equipment (PPE)
  ○ It is vital to **____** the patient as safely and quickly as possible
    ▪ Answer: decontaminate
  ○ First, remove the patient from the **____** and remove contaminated clothing
    ▪ Answer: source
  ○ Then, ensure the skin is washed with **____ ____ ____**
    ▪ Answer: soap and water
  ○ Finally, ensure there is proper **____** of both the contaminated clothing, as well as the water run-off from the cleaning of the patient.
    ▪ Answer: disposal
- Key to next clue: “You reach for the bag valve mask” statement in the final slide as well as a photo of personal in PPE bagging a patient.

**Clue 8 (Bag valve mask)**

- Crossword puzzle. Click on each column and row to view the clue and type in the answer.
  ○ Avoid intubation with this medication because it is metabolized by acetylcholinesterase and may cause prolonged paralysis
    ▪ Answer: succinylcholine
  ○ Hypoxia and wheezing secondary to muscarinic toxicity will only definitively respond to the antidote
    ▪ Answer: atropine
  ○ Nondepolarizing neuromuscular blocking agents, like rocuronium, may be less effective at standard dosing given competition inhibiting which junction?
    ▪ Answer: neuromuscular
  ○ Respiratory failure is due to a combination of effects, including central nervous system depression, neuromuscular weakness, excessive respiratory secretions and *******
    ▪ Answer: bronchoconstriction
  ○ Supplemental **** may be minimally beneficial for hypoxia
    ▪ Answer: oxygen
- Key to next clue: “syringe” is highlighted throughout the crossword puzzle when complete.[Fig f10-9-3-sg36]

**Clue 9 (Syringe)**

- Use a combination lock to unlock the medication drawer and get further doses of the antidote to “escape the room” and ultimately, save the patient.
- What is the initial dosage of atropine (in mg) for this patient?
  ○ Based on Broselow tape (kg= 30) with initial dosage of 0.05mg/kg
  ○ Answer: 1.5mg
- They escaped![Fig f11-9-3-sg36]

### Appendix B Key Clinical Pearls

**Epidemiology and etiology**

- Worldwide, an average of 3 million people are exposed each year, resulting on average of 300,000 deaths^14^,^15^
- Toxicity usually is secondary to accidental/intentional ingestion of or exposure to agricultural pesticides.^16^–^18^
- Other potential causes can be ingestion of contaminated food or wearing contaminated clothing.
- Has been used as chemical terrorism in times of war.^14^,^16^

**Mechanism of Action**

- They are well absorbed through the skin, lungs, and gastrointestinal tract.^14^–^18^
- They bind to acetylcholinesterase (AchE) or red blood cell acetylcholinesterase, inhibiting the enzymatic activity.^14^–^16^,^19^
  ○ Acetylcholine is the neurotransmitter broken down by AchE.
  ○ The inhibition leads to an abundance of acetylcholine at the synapse and the neuromuscular junction leading to an increase of activity at the receptors.
- After a period of time, the ongoing binding of AchE to organophosphates creates a conformational change, rendering the enzyme irreversible to reactivation-also known as aging.^14^,^16^

**Clinical Presentation**^14^–^16^,^19^

- Onset and duration of symptoms depend on the agent itself, and the route of absorption.^19^
  ○ Oral and respiratory exposure can result in symptoms within a few hours
  ○ Skin exposure can be delayed up to 12 hours
  ○ All routes of absorption affect both muscarinic and nicotinic receptors
- Presentation represents cholinergic excess from persistent exposure to acetylcholine at the synapse.^14^,^16^
  ○ DUMBELS: Defecation, Urination, Miosis, Bronchorrhea/bronchospasm/bradycardia, Emesis, Lacrimation, Salivation
- However, acetylcholine is a neurotransmitter not only for the autonomic nervous system, but for the central nervous system and neuromuscular junction as well.
- There are nicotinic receptors at the neuromuscular junction where acetylcholine is a key neurotransmitter.^19^
  ○ This can lead to fasciculations, muscle weakness, and paralysis
- There are nicotinic and muscarinic receptors in the central nervous system.^19^
  ○ This can lead to coma, seizures, lethargy, and respiratory depression
- Respiratory failure can be a combination of CNS depression, neuromuscular weakness, excessive secretions and bronchoconstriction.^14^

**Workup**

- Diagnosis usually based on clinical findings and clinical features of cholinergic excess, even in the absence of a known exposure or ingestion.
- Can have garlic-like or petroleum odor.^14^
- RBC AchE provides a measurement of the level of toxicity and can help guide response to therapy.^14^
  ○ Can take time to obtain and result, and this should not delay treatment
- Chest x-ray may be clear to severe cases with pulmonary edema.
- ECG classically will show sinus bradycardia, but can have ventricular dysrhythmia, prolonged QTc, torsades de pointes.^14^,^16^
- Laboratory evaluation can show evidence of elevated amylase, leukocytosis, hemoconcentration, hypokalemia, hypomagnesemia, elevated LFT, elevated troponin, metabolic and/or respiratory acidosis.^14^

**Management**

- Any person coming in contact with the patient should be in PPE because toxin is readily absorbed.
- The patient should be decontaminated as quickly and safely as possible.^14^,^16^
  ○ Remove them from the source
  ○ Remove the contaminated clothing
  ○ Wash the skin with copious amounts of soap and water, with proper disposal of the run off
- Adequate volume resuscitation with isotonic fluids should be performed throughout management.
- Seizures should be treated with benzodiazepine.
- Patients with severely depressed mental status should be given oxygen and intubated given risk for respiratory failure.
  ○ Most common cause of death in organophosphate poisoning is respiratory failure^14^,^16^
  ○ Avoid use of succinylcholine for rapid sequence intubation because it is metabolized by AchE, which is inhibited by organophosphates. This may lead to prolonged neuromuscular blockade^19^
  ○ Increased dosages of nondepolarizing neuromuscular blocking agents may need to be used due to competition at the neuromuscular junction^14^
- Cholinergic toxicity is managed with atropine and pralidoxime (2-PAM).
- Atropine^14^–^16^,^19^
  ○ Competes with Ach at the muscarinic receptors
  ○ Initial dosage: 0.02–0.05mg/kg via IV
  ○ Double the dosage every 3–5 minutes until pulmonary muscarinic signs are alleviated
- 2-PAM^14^–^16^,^19^
  ○ Necessary because atropine does not bind to nicotinic receptors and will not treat neuromuscular dysfunction
  ○ Reversibly binds to AchE, competing with organophosphate binding
  ○ Should be given as early as possible to prevent permanent inactivation of the enzyme (aging)
  ○ 20–50 mg/kg IV, based on the severity of symptoms
  ○ Infuse slowly over 30 minutes
    ▪ Rapid administration can be associated with cardiac arrest
  Atropine must be given before 2-PAM to avoid worsening of muscarinic effect

### Appendix C Formal Presentation

[Fig f12-9-3-sg36]

### Appendix D Virtual Escape Room Survey

1. I identify as
  - Male
  - Female
  - Nonbinary
2. Year in training
  - PGY-1
  - PGY-2
  - PGY-3
3. I have participated in an escape room in the past
  - Yes
  - No
4. If yes, was this for recreation or education?
5. I have participated in a virtual escape room in the past
  - Yes
  - No
6. If yes, was this for recreation or education?[Table t4-9-3-sg36]
  **Table t4-9-3-sg36:**
  	Strongly disagree	Disagree	Neither agree nor disagree	Agree	Strongly agree
  The design was easy to follow					
  This was an engaging educational model					
  The addition of puzzles did not take away from the patient case					
  This educational model created a stressful environment					
  I would prefer virtual escape rooms over traditional didactics					
  This was a feasible model to reinforce medical knowledge					
  I was able to identify my gaps in knowledge with this case					
  This utilized critical thinking skills to solve puzzles					
  Completing the puzzles facilitated communication between team members					
  I would participate in a virtual escape room in the future					
7. What did you enjoy about this activity?
8. What would you change about this activity?
9. Reflection: How will you apply what you learned from this session in clinical practice?
