# Supplementary material for: Development and Design of a Pediatric Case-Based Virtual Escape Room on Organophosphate Toxicity
Source: J Educ Teach Emerg Med. 2024 Jul 31;9(3):SG36–62. doi: 10.21980/J8DH1V (PMC11312879; doi:10.21980/J8DH1V)
Supplement: Supplementary file 1 [file 9-3-SG36-Supp1.pptx]

## Slide 1
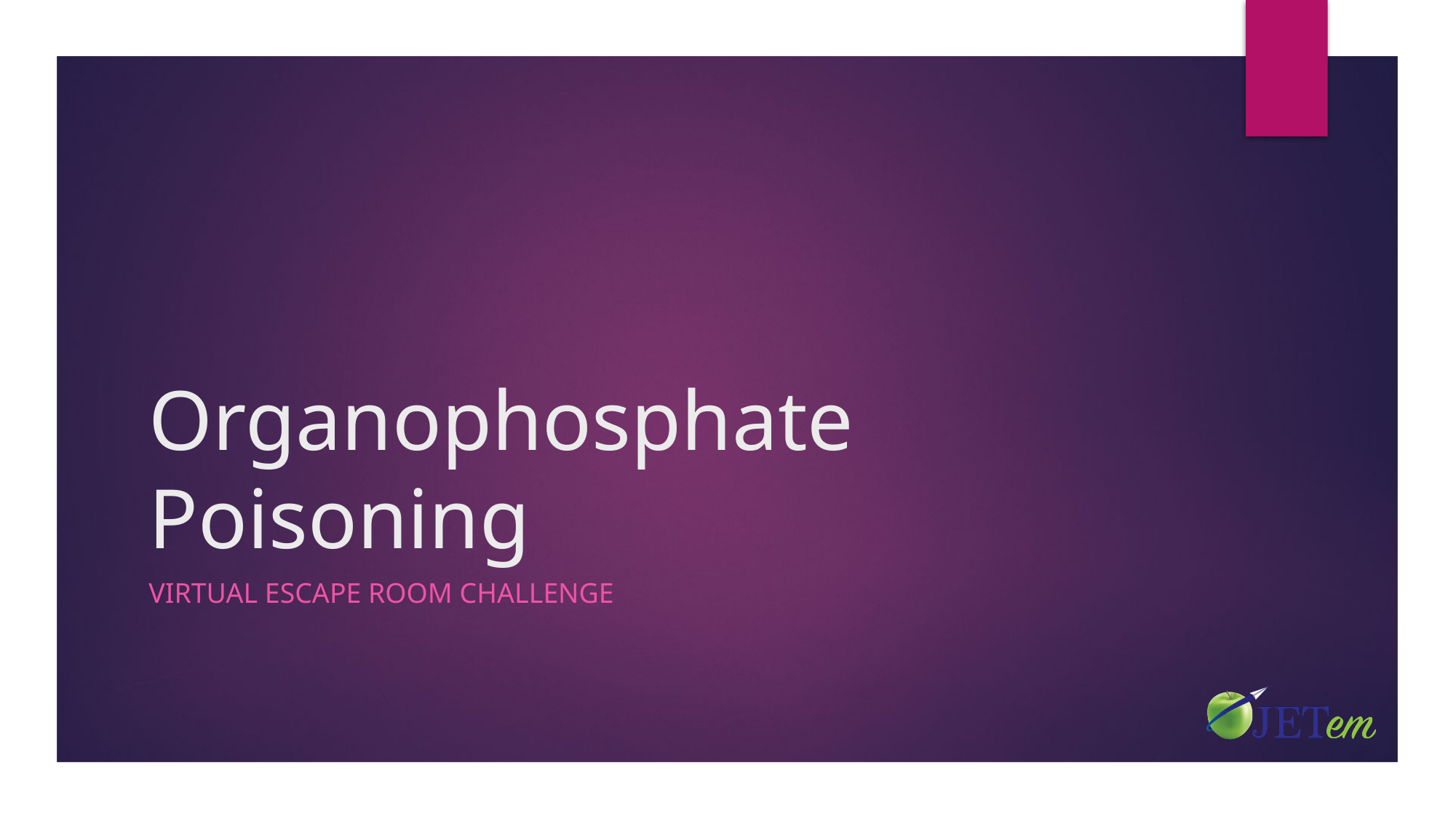

# Organophosphate Poisoning
Virtual Escape Room Challenge

## Slide 2
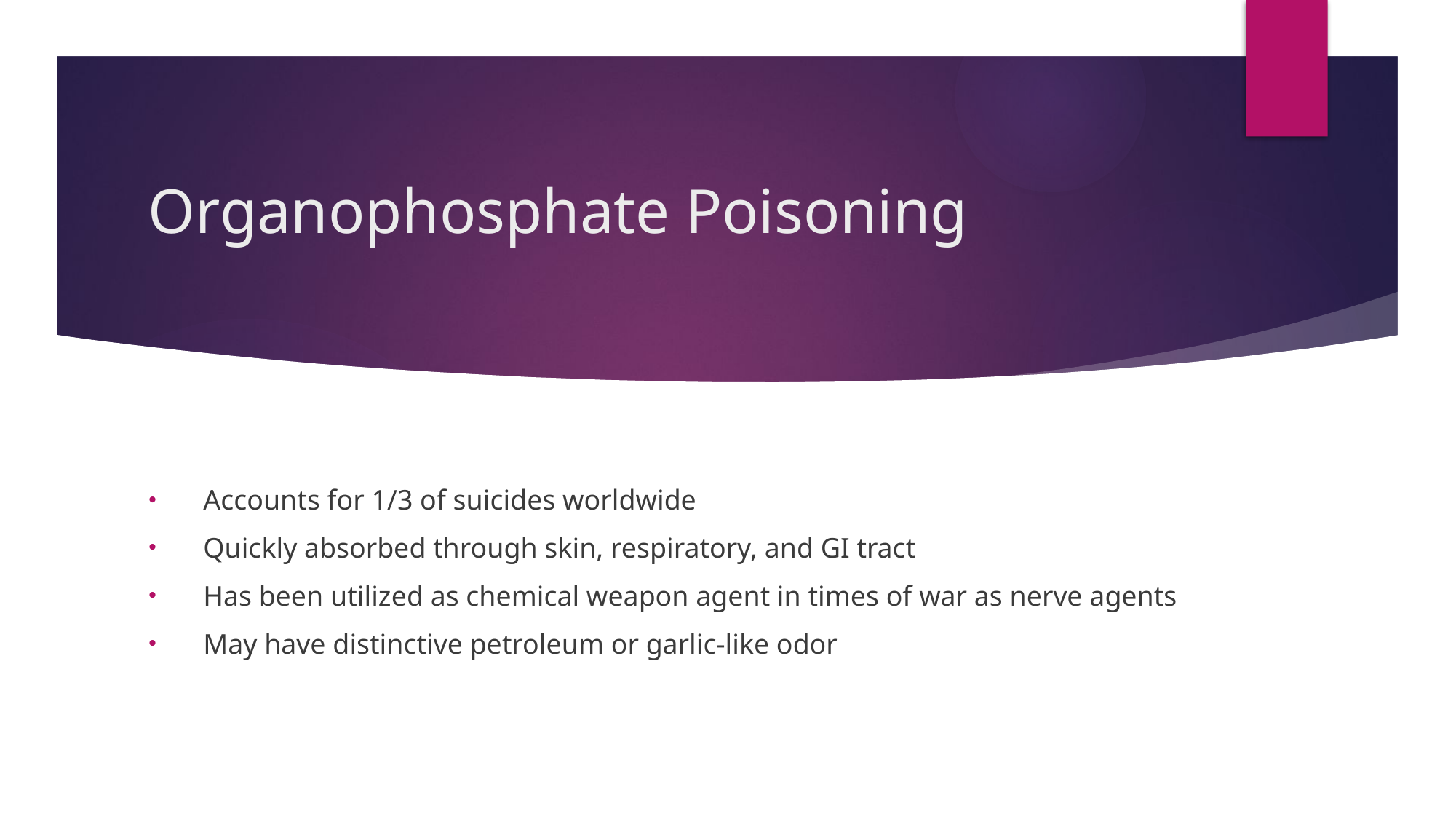

# Organophosphate Poisoning
Accounts for 1/3 of suicides worldwide
Quickly absorbed through skin, respiratory, and GI tract
Has been utilized as chemical weapon agent in times of war as nerve agents
May have distinctive petroleum or garlic-like odor

## Slide 3
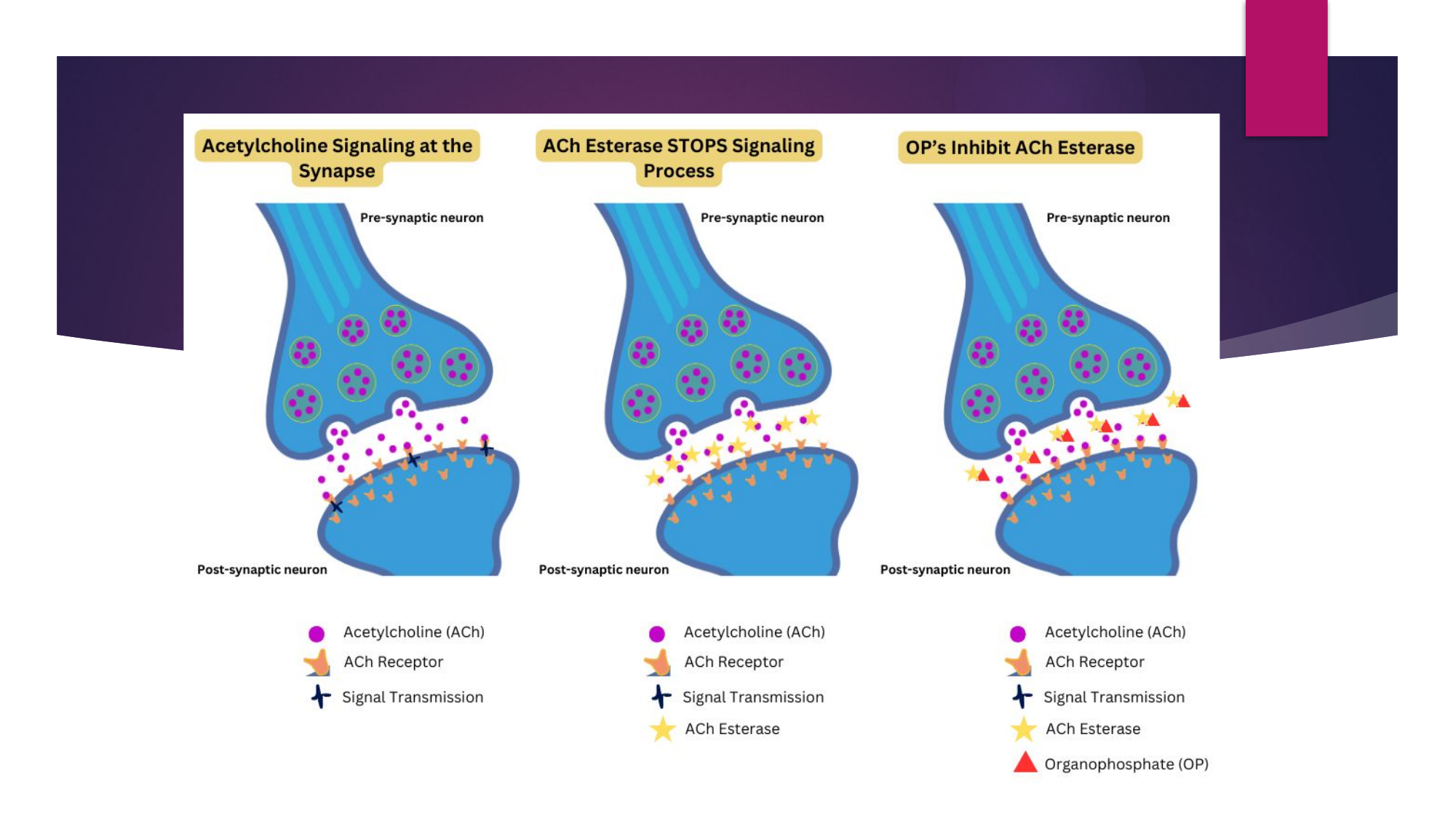

## Slide 4
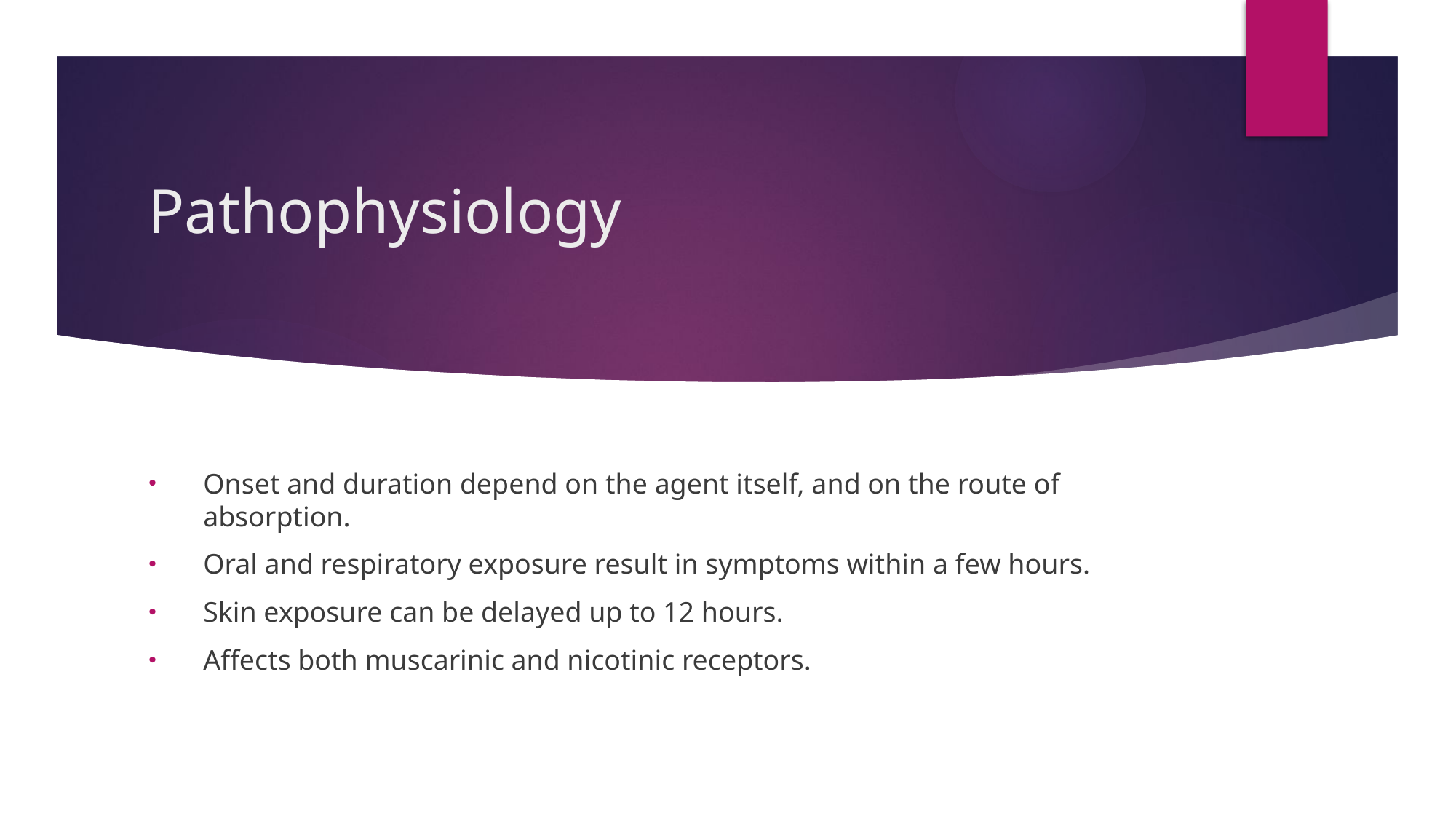

# Pathophysiology
Onset and duration depend on the agent itself, and on the route of absorption.
Oral and respiratory exposure result in symptoms within a few hours.
Skin exposure can be delayed up to 12 hours.
Affects both muscarinic and nicotinic receptors.

## Slide 5
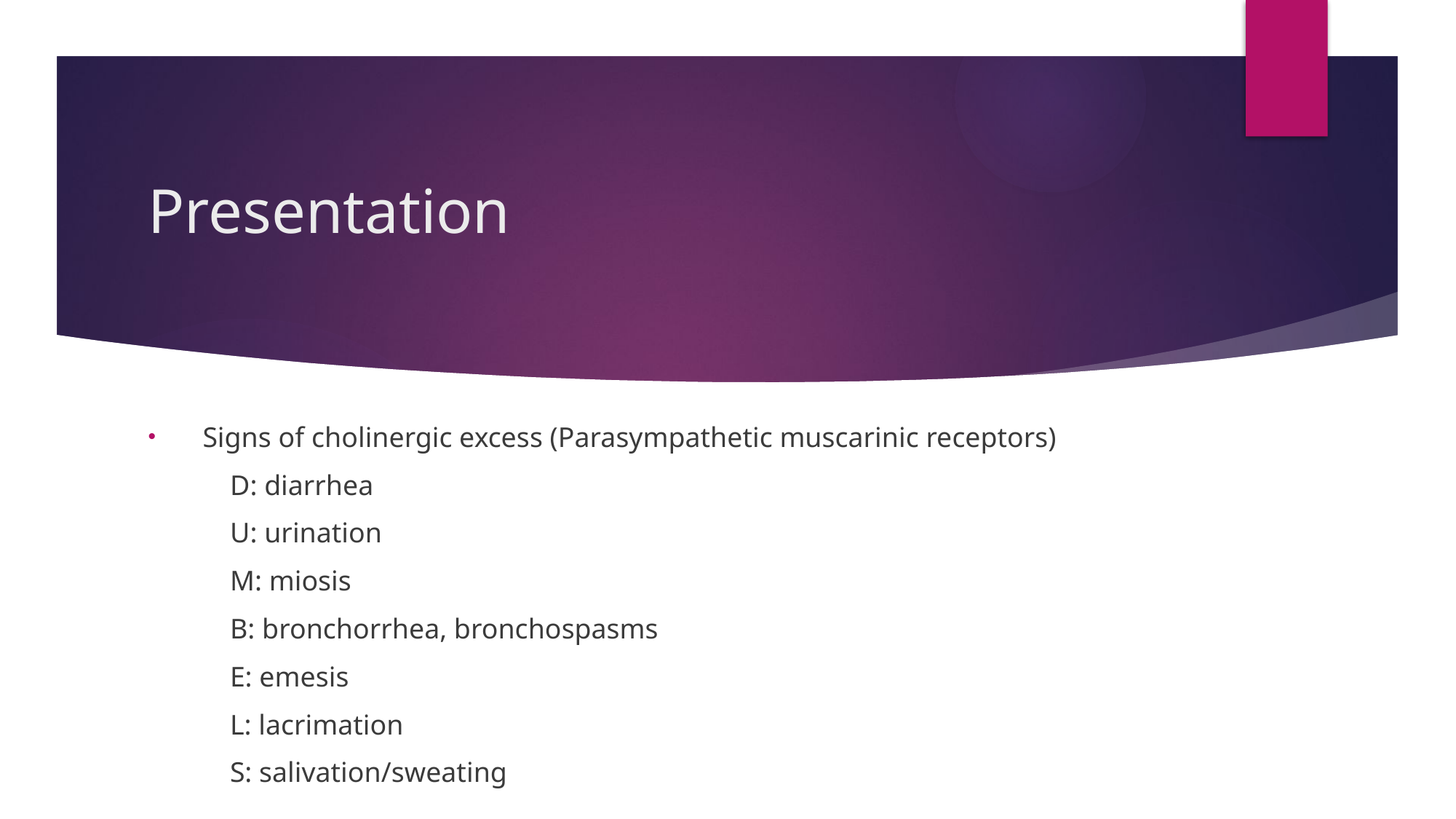

# Presentation
Signs of cholinergic excess (Parasympathetic muscarinic receptors)
D: diarrhea
U: urination
M: miosis
B: bronchorrhea, bronchospasms
E: emesis
L: lacrimation
S: salivation/sweating

## Slide 6
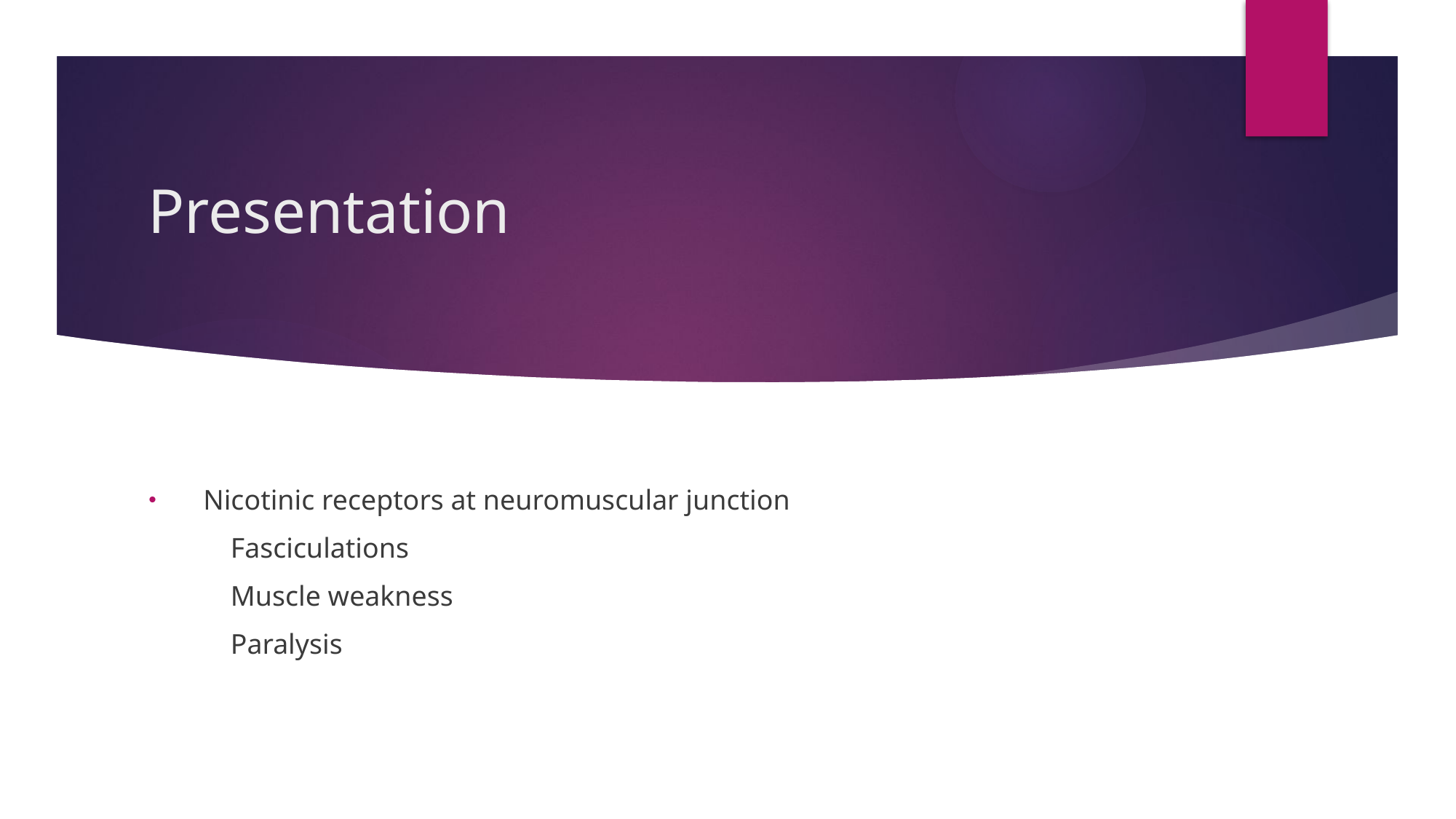

# Presentation
Nicotinic receptors at neuromuscular junction
Fasciculations
Muscle weakness
Paralysis

## Slide 7
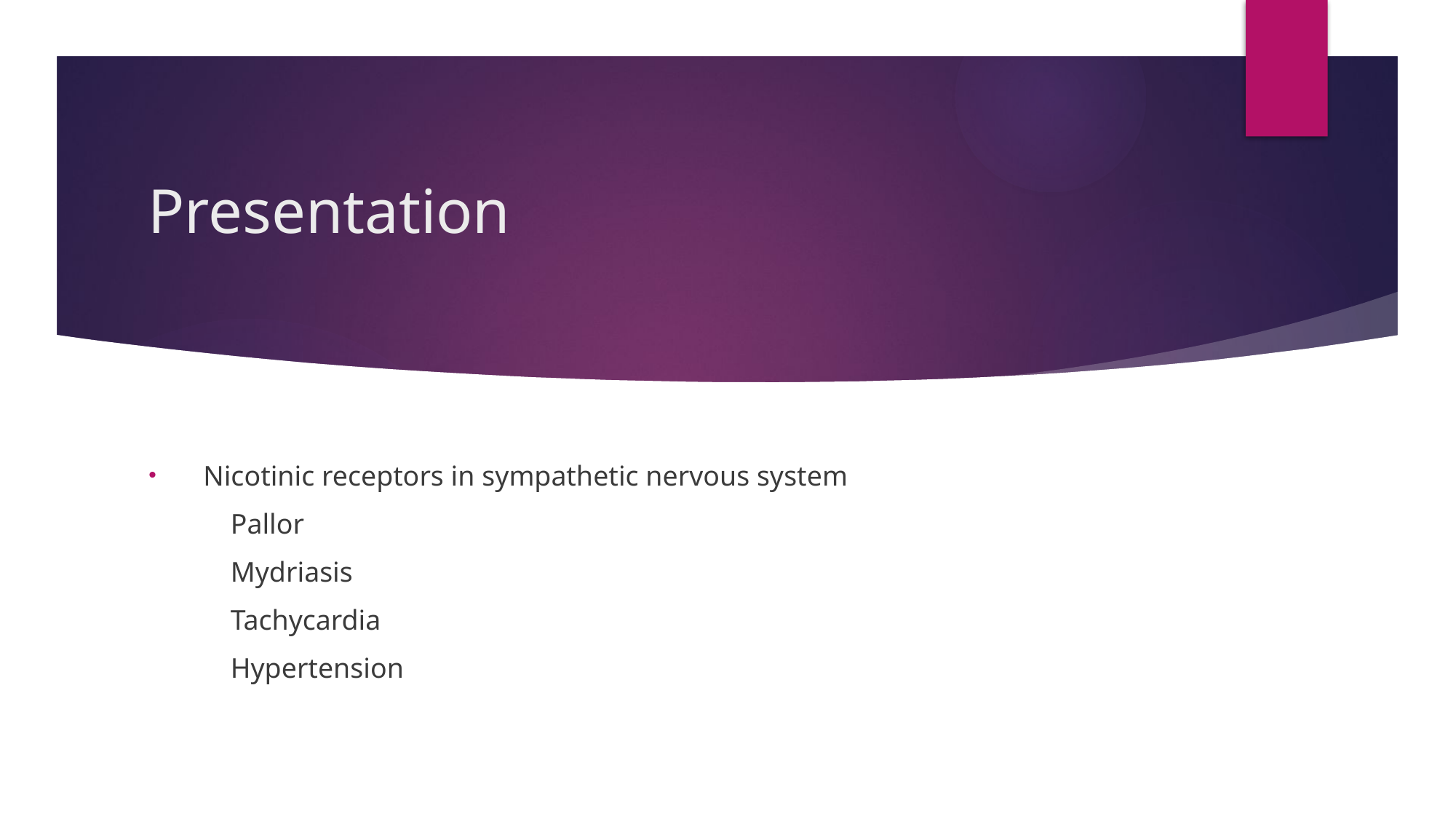

# Presentation
Nicotinic receptors in sympathetic nervous system
Pallor
Mydriasis
Tachycardia
Hypertension

## Slide 8
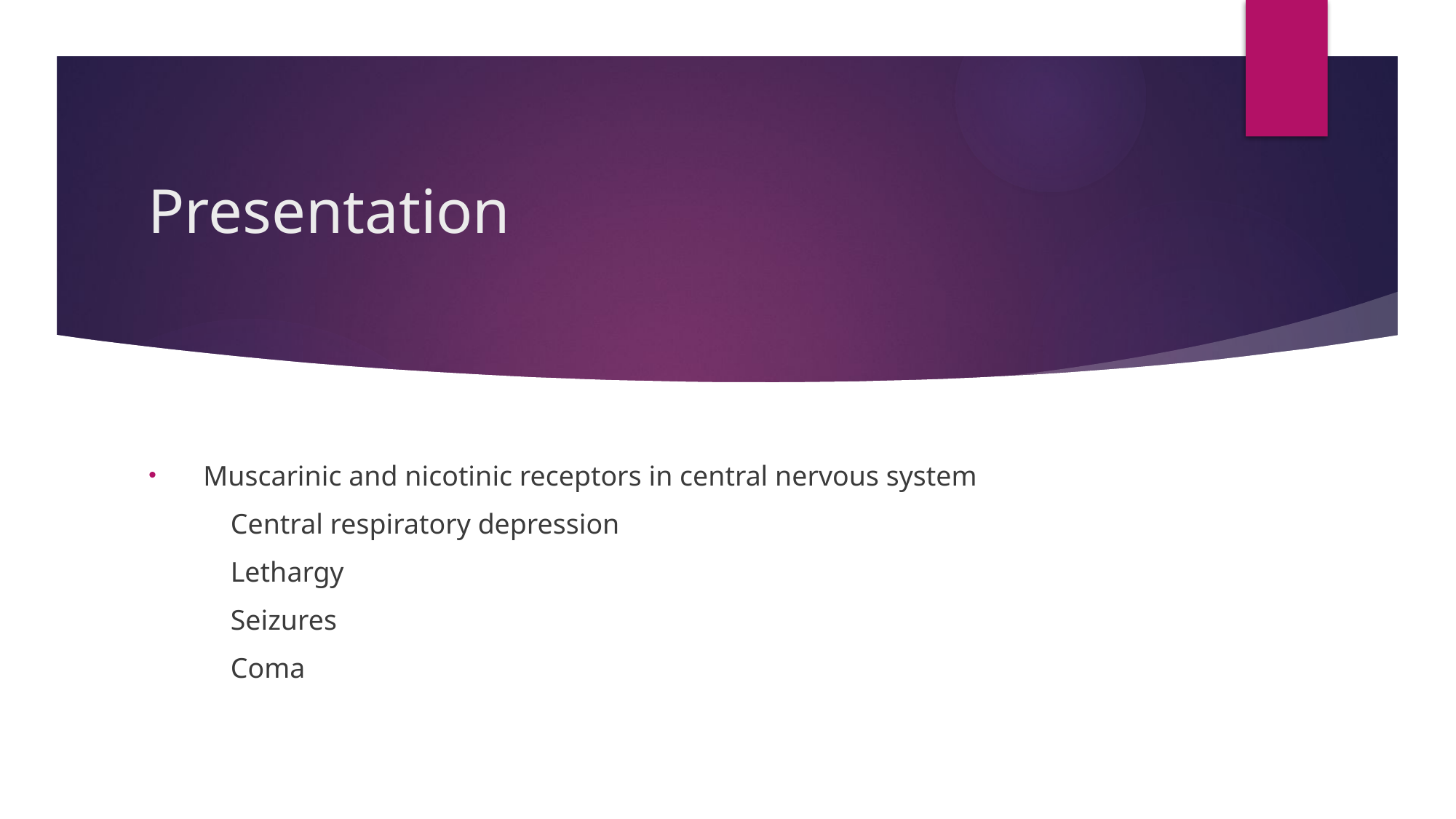

# Presentation
Muscarinic and nicotinic receptors in central nervous system
Central respiratory depression
Lethargy
Seizures
Coma

## Slide 9
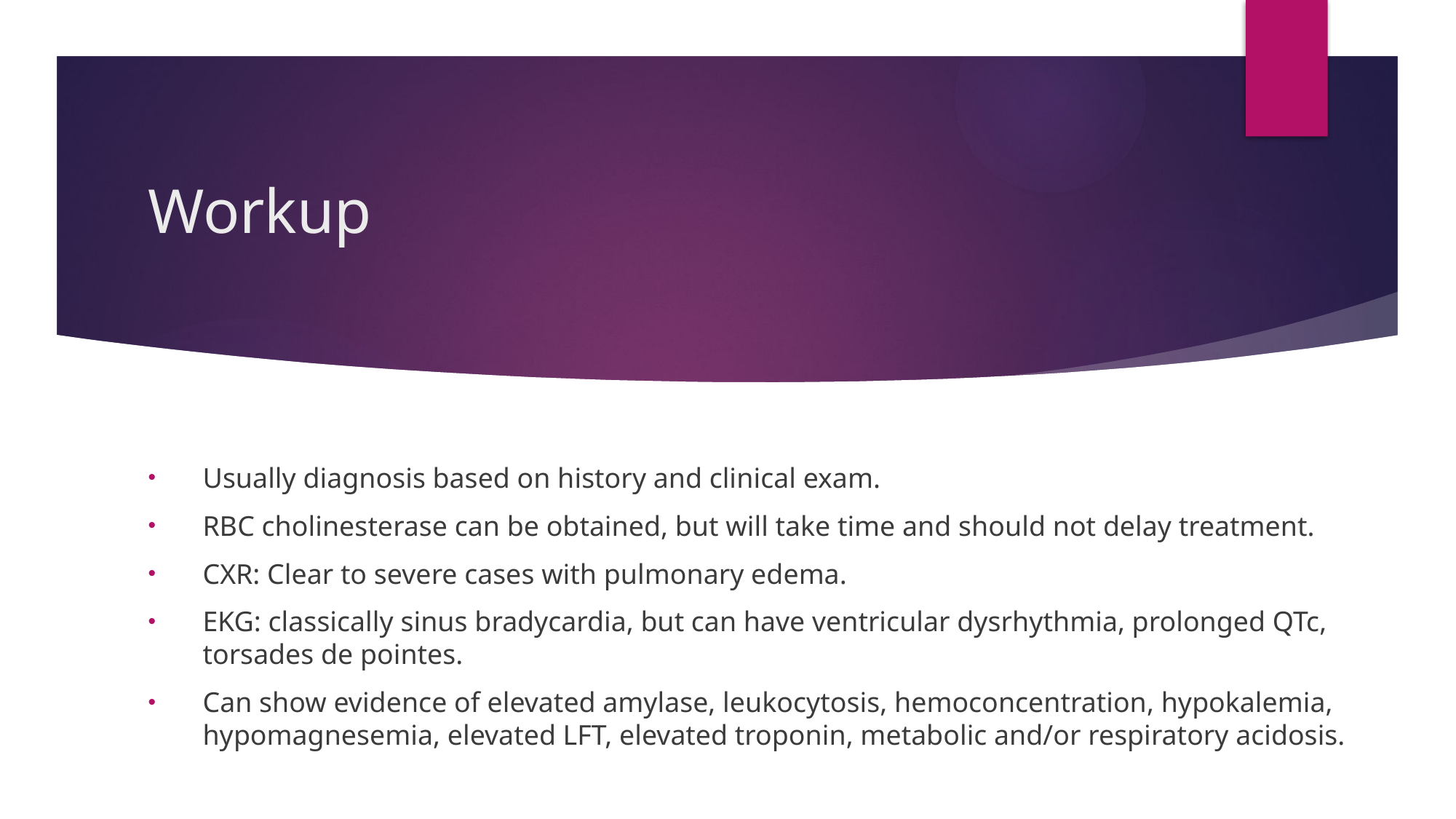

# Workup
Usually diagnosis based on history and clinical exam.
RBC cholinesterase can be obtained, but will take time and should not delay treatment.
CXR: Clear to severe cases with pulmonary edema.
EKG: classically sinus bradycardia, but can have ventricular dysrhythmia, prolonged QTc, torsades de pointes.
Can show evidence of elevated amylase, leukocytosis, hemoconcentration, hypokalemia, hypomagnesemia, elevated LFT, elevated troponin, metabolic and/or respiratory acidosis.

## Slide 10
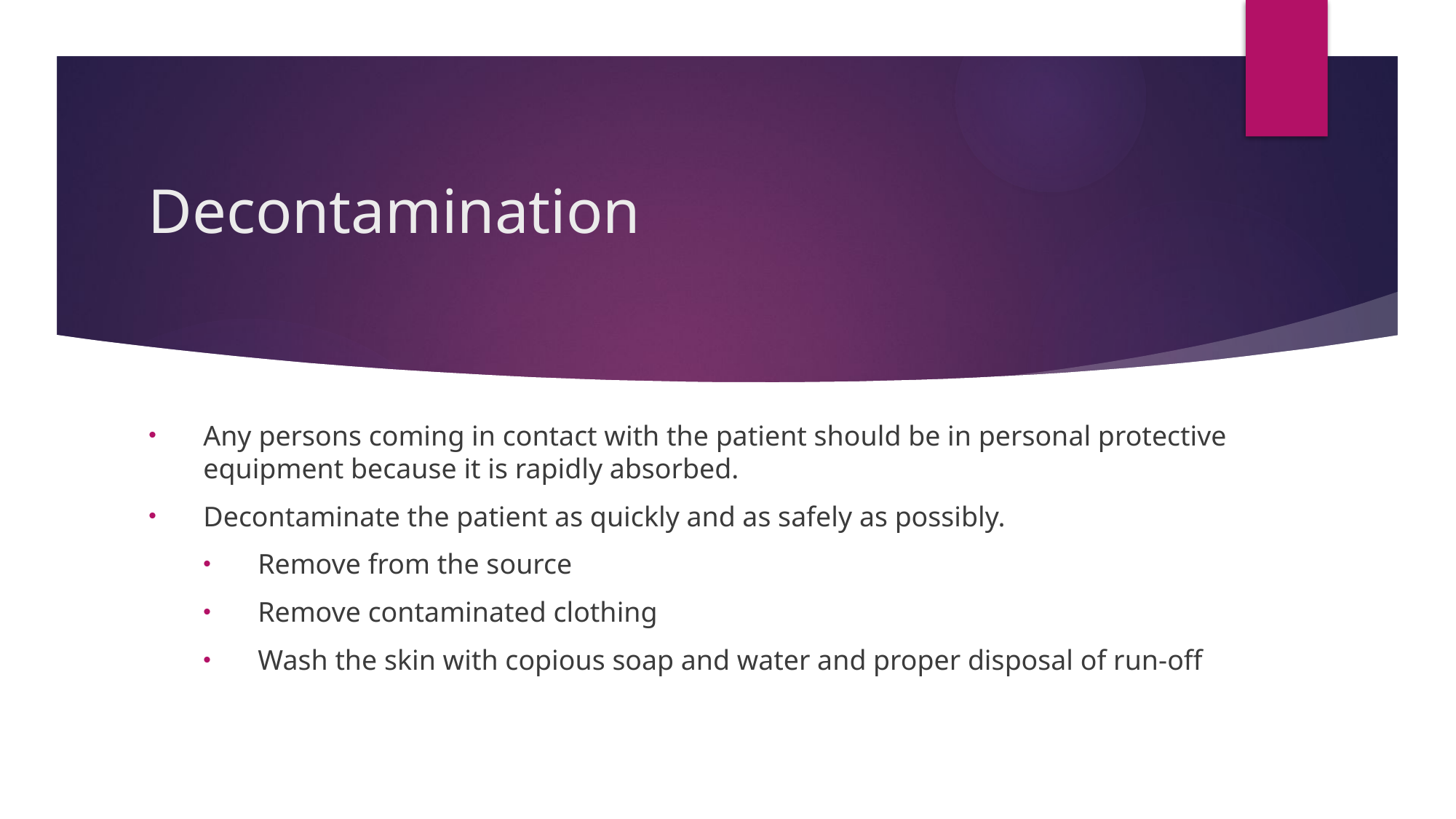

# Decontamination
Any persons coming in contact with the patient should be in personal protective equipment because it is rapidly absorbed.
Decontaminate the patient as quickly and as safely as possibly.
Remove from the source
Remove contaminated clothing
Wash the skin with copious soap and water and proper disposal of run-off

## Slide 11
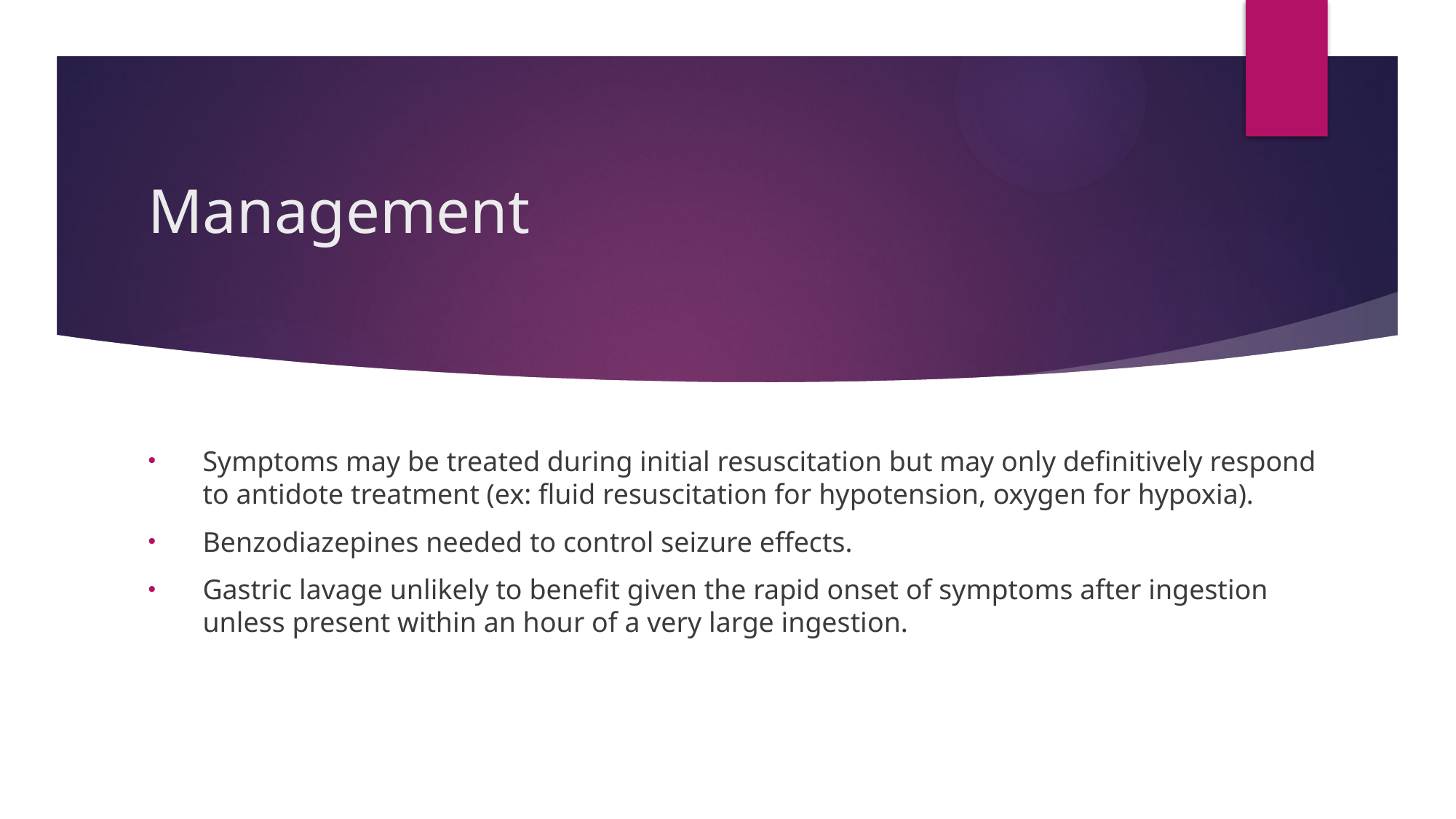

# Management
Symptoms may be treated during initial resuscitation but may only definitively respond to antidote treatment (ex: fluid resuscitation for hypotension, oxygen for hypoxia).
Benzodiazepines needed to control seizure effects.
Gastric lavage unlikely to benefit given the rapid onset of symptoms after ingestion unless present within an hour of a very large ingestion.

## Slide 12
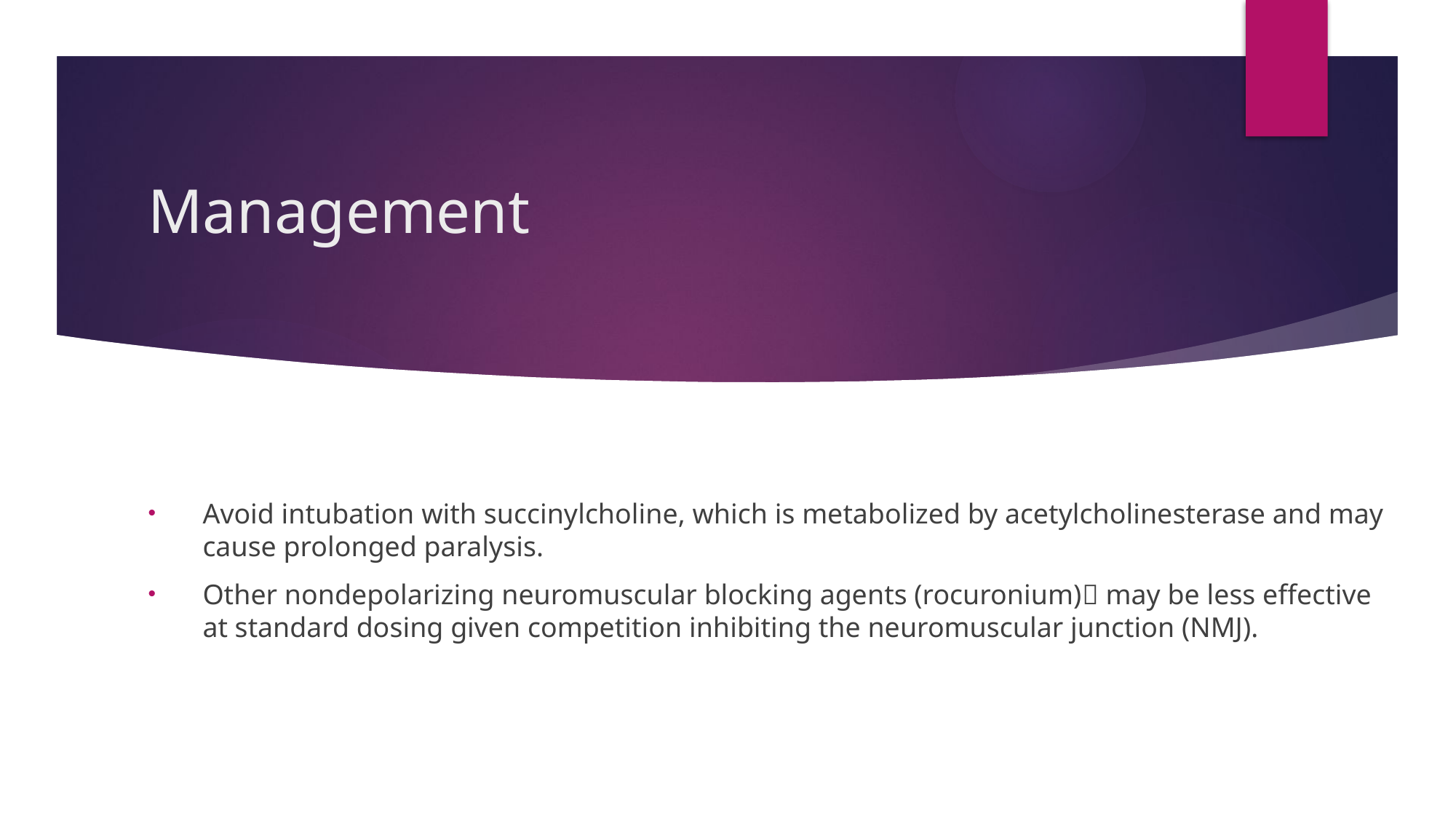

# Management
Avoid intubation with succinylcholine, which is metabolized by acetylcholinesterase and may cause prolonged paralysis.
Other nondepolarizing neuromuscular blocking agents (rocuronium) may be less effective at standard dosing given competition inhibiting the neuromuscular junction (NMJ).

## Slide 13
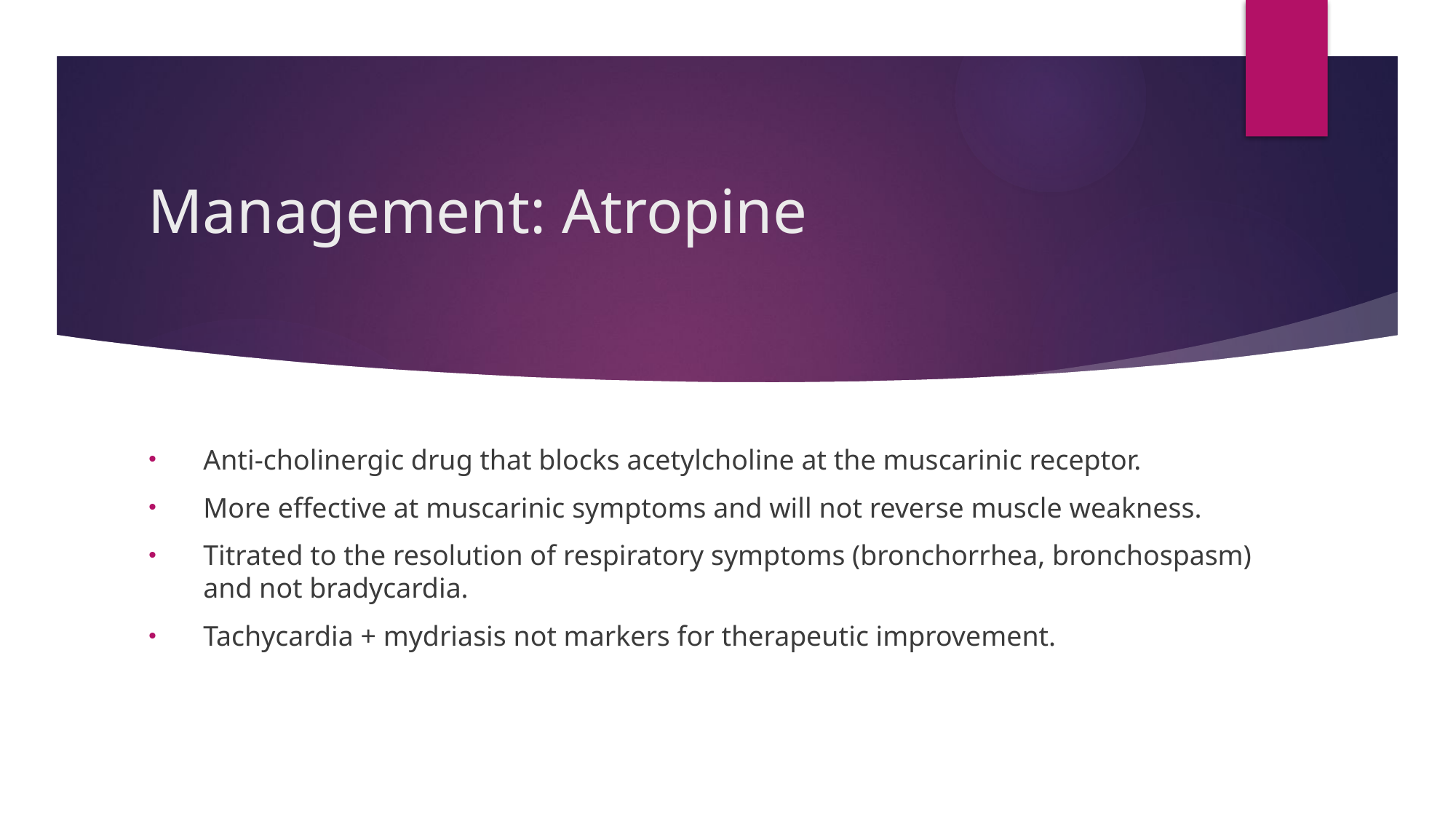

# Management: Atropine
Anti-cholinergic drug that blocks acetylcholine at the muscarinic receptor.
More effective at muscarinic symptoms and will not reverse muscle weakness.
Titrated to the resolution of respiratory symptoms (bronchorrhea, bronchospasm) and not bradycardia.
Tachycardia + mydriasis not markers for therapeutic improvement.

## Slide 14
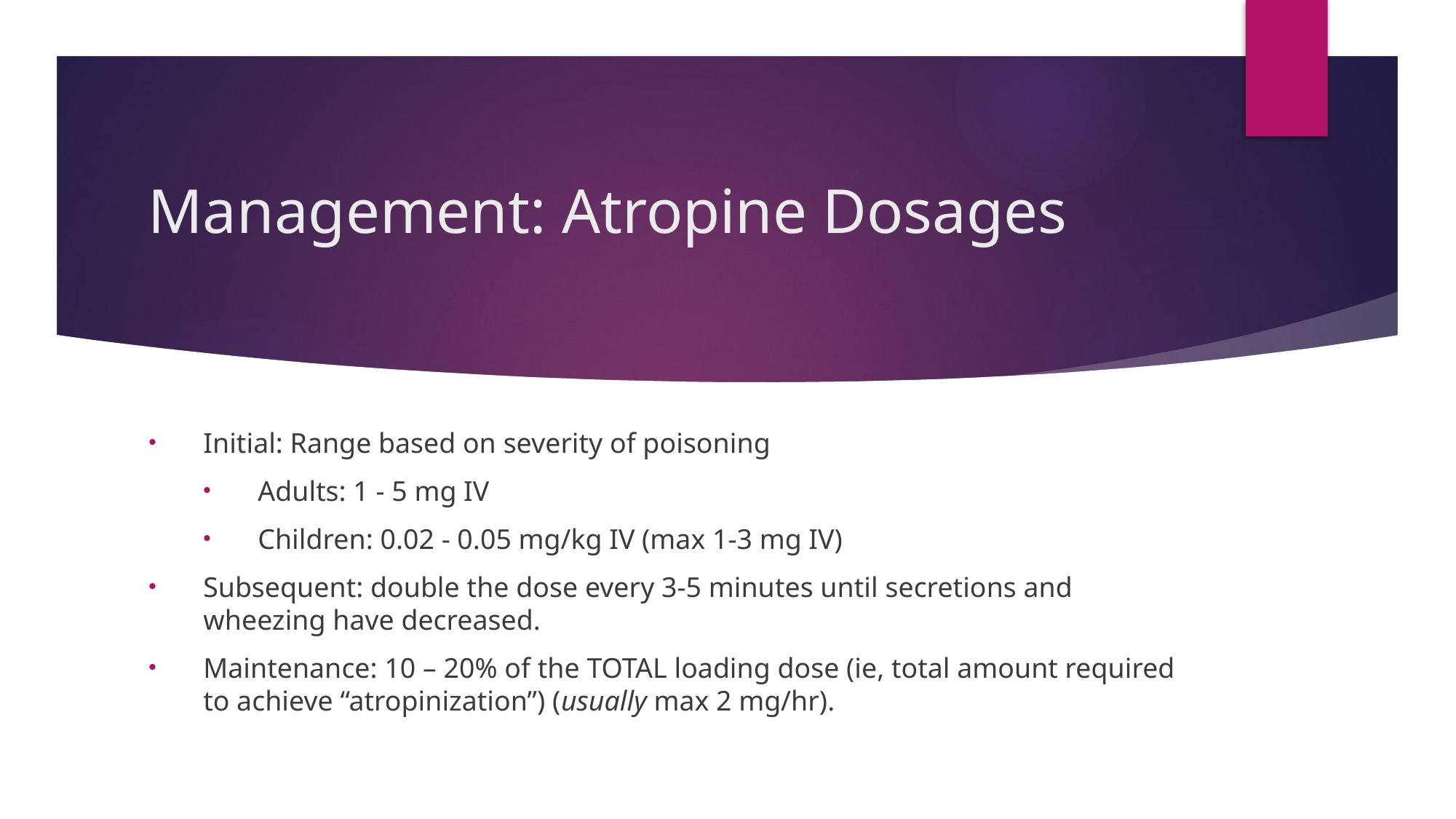

# Management: Atropine Dosages
Initial: Range based on severity of poisoning
Adults: 1 - 5 mg IV
Children: 0.02 - 0.05 mg/kg IV (max 1-3 mg IV)
Subsequent: double the dose every 3-5 minutes until secretions and wheezing have decreased.
Maintenance: 10 – 20% of the TOTAL loading dose (ie, total amount required to achieve “atropinization”) (usually max 2 mg/hr).

## Slide 15
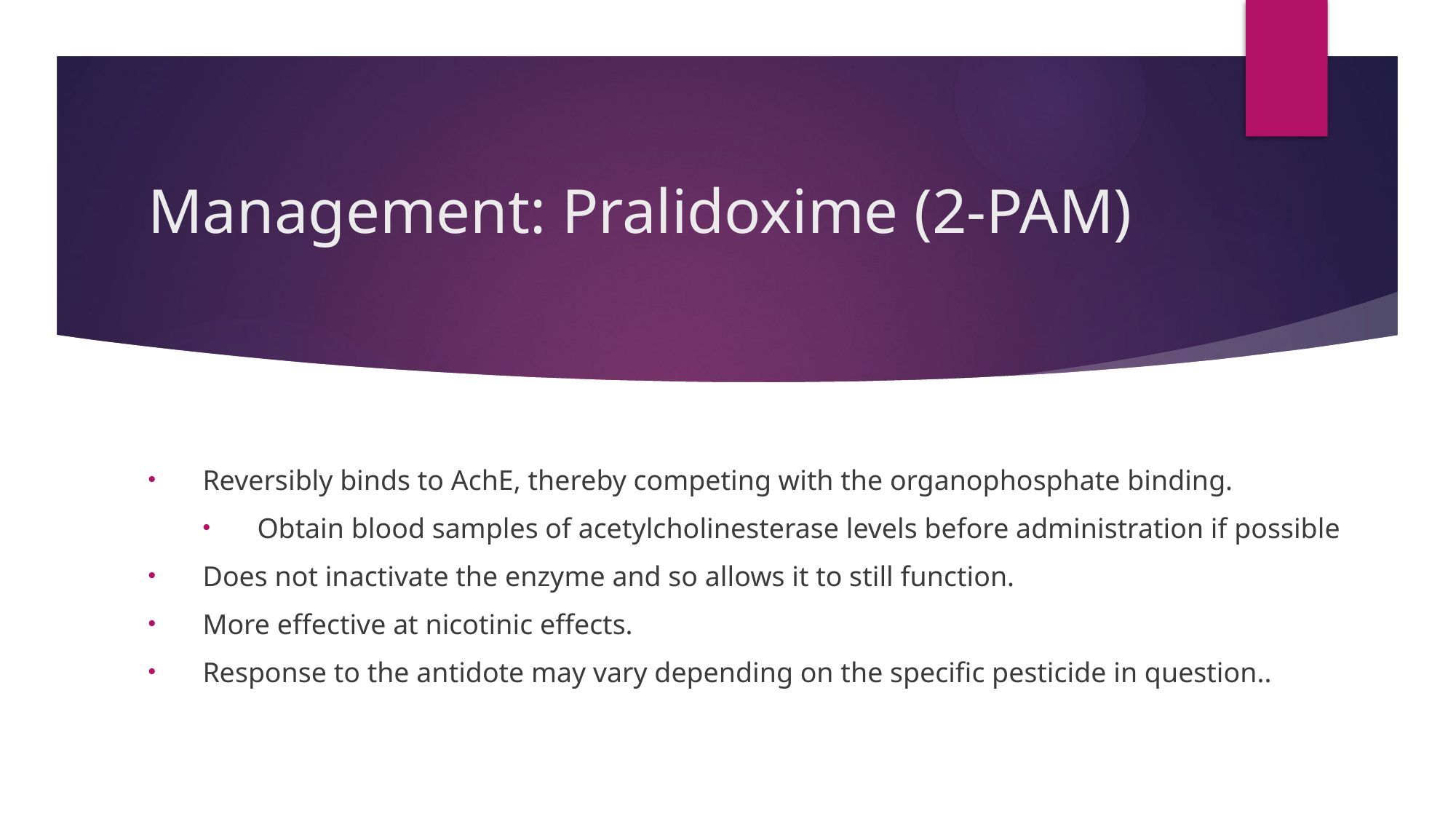

# Management: Pralidoxime (2-PAM)
Reversibly binds to AchE, thereby competing with the organophosphate binding.
Obtain blood samples of acetylcholinesterase levels before administration if possible
Does not inactivate the enzyme and so allows it to still function.
More effective at nicotinic effects.
Response to the antidote may vary depending on the specific pesticide in question..

## Slide 16
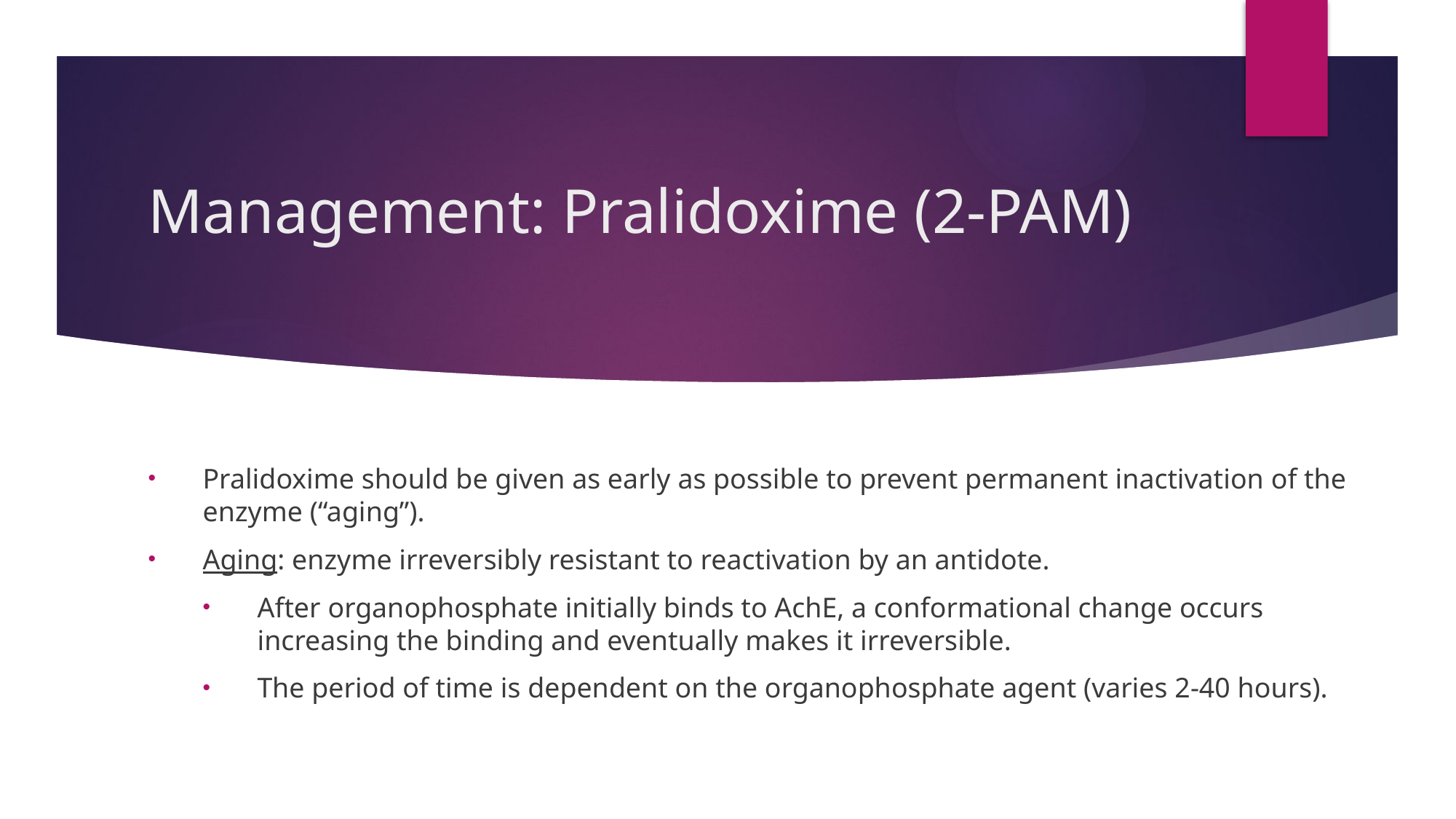

# Management: Pralidoxime (2-PAM)
Pralidoxime should be given as early as possible to prevent permanent inactivation of the enzyme (“aging”).
Aging: enzyme irreversibly resistant to reactivation by an antidote.
After organophosphate initially binds to AchE, a conformational change occurs increasing the binding and eventually makes it irreversible.
The period of time is dependent on the organophosphate agent (varies 2-40 hours).

## Slide 17
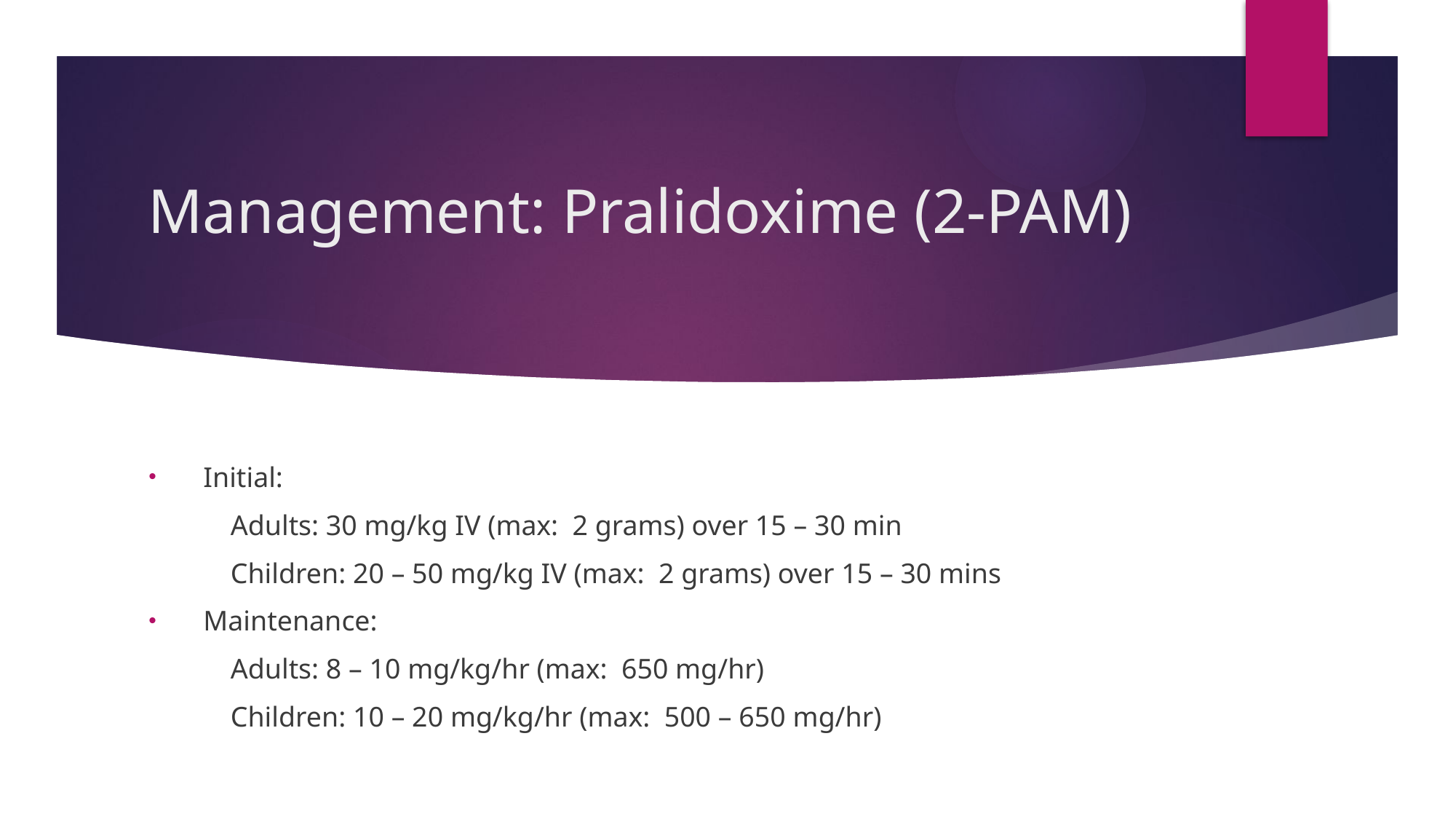

# Management: Pralidoxime (2-PAM)
Initial:
Adults: 30 mg/kg IV (max:  2 grams) over 15 – 30 min
Children: 20 – 50 mg/kg IV (max:  2 grams) over 15 – 30 mins
Maintenance:
Adults: 8 – 10 mg/kg/hr (max:  650 mg/hr)
Children: 10 – 20 mg/kg/hr (max:  500 – 650 mg/hr)

## Slide 18
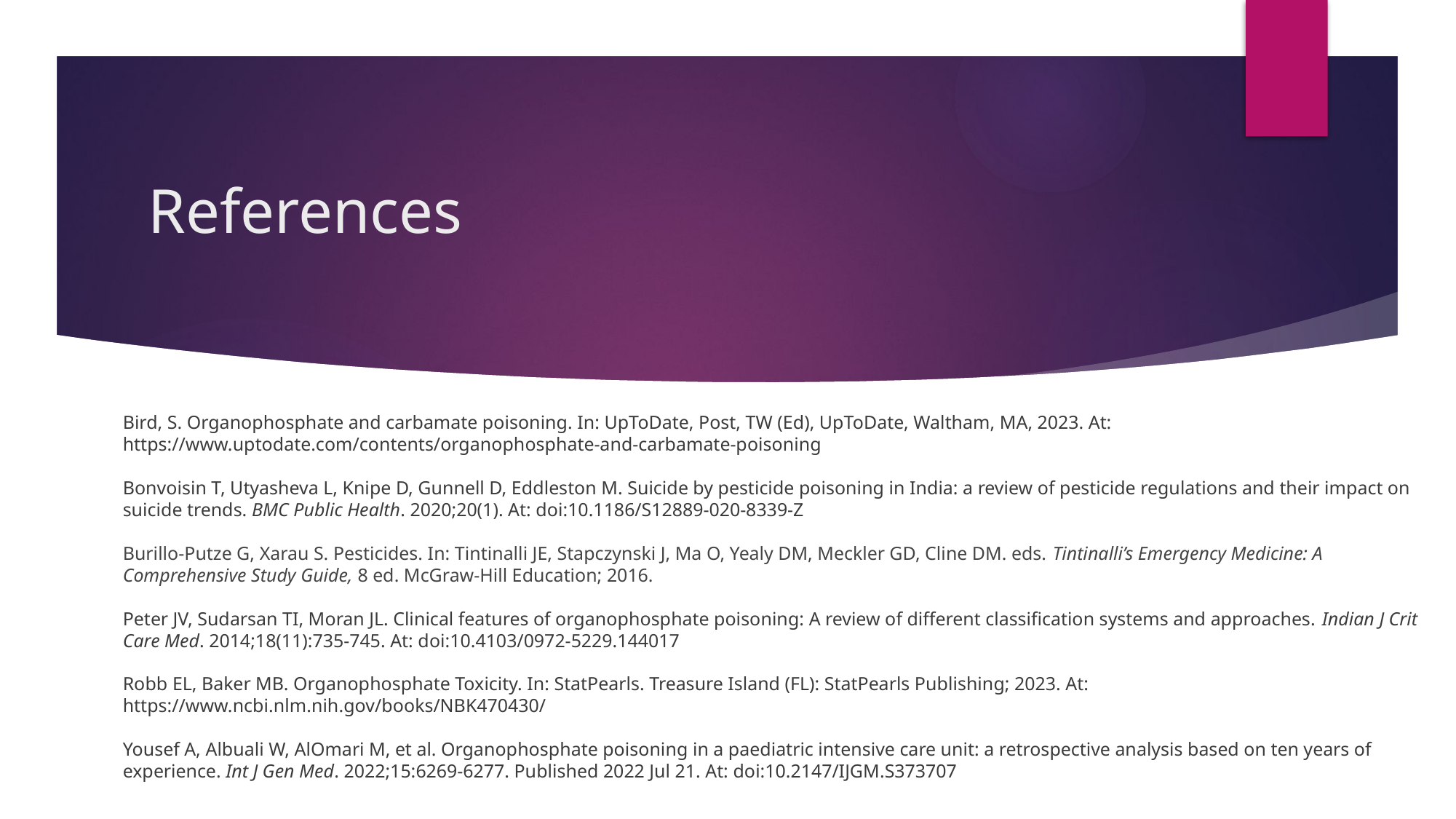

# References
Bird, S. Organophosphate and carbamate poisoning. In: UpToDate, Post, TW (Ed), UpToDate, Waltham, MA, 2023. At: https://www.uptodate.com/contents/organophosphate-and-carbamate-poisoning
Bonvoisin T, Utyasheva L, Knipe D, Gunnell D, Eddleston M. Suicide by pesticide poisoning in India: a review of pesticide regulations and their impact on suicide trends. BMC Public Health. 2020;20(1). At: doi:10.1186/S12889-020-8339-Z
Burillo-Putze G, Xarau S. Pesticides. In: Tintinalli JE, Stapczynski J, Ma O, Yealy DM, Meckler GD, Cline DM. eds. Tintinalli’s Emergency Medicine: A Comprehensive Study Guide, 8 ed. McGraw-Hill Education; 2016.
Peter JV, Sudarsan TI, Moran JL. Clinical features of organophosphate poisoning: A review of different classification systems and approaches. Indian J Crit Care Med. 2014;18(11):735-745. At: doi:10.4103/0972-5229.144017
Robb EL, Baker MB. Organophosphate Toxicity. In: StatPearls. Treasure Island (FL): StatPearls Publishing; 2023. At: https://www.ncbi.nlm.nih.gov/books/NBK470430/
Yousef A, Albuali W, AlOmari M, et al. Organophosphate poisoning in a paediatric intensive care unit: a retrospective analysis based on ten years of experience. Int J Gen Med. 2022;15:6269-6277. Published 2022 Jul 21. At: doi:10.2147/IJGM.S373707
